# The screening and functional study of proteins binding with the BmNPV polyhedrin promoter

**DOI:** 10.1186/1743-422X-9-90

**Published:** 2012-05-06

**Authors:** Wei Yu, Jia Li, Meihui Wang, Yanping Quan, Jian Chen, Zuoming Nie, Zhengbing Lv, Yaozhou Zhang

**Affiliations:** 1 Institute of Biochemistry, Zhejiang Sci-Tech University, Hangzhou, China; 2 Zhejiang Provincial Key Laboratory of Silkworm Bioreactor and Biomedicine, Hangzhou, China

## Abstract

**Background:**

The polyhedrin gene promoter has an essential role in regulating foreign gene expression in baculovirus expression vector systems (BEVS); however, the high-level transcription mechanism is still unknown. One-hybrid screening in yeast is a powerful way of identifying rapidly heterologous transcription factors that can interact with the polyhedrin promoter DNA sequence. In the current study, total RNA was extracted from the fat bodies of fifth-instar silkworm larvae that had been infected with *Bombyx mori* nuclear polyhedrosis virus (BmNPV) for 5 days; complementary DNA (cDNA) was then generated using reverse-transcription (RT)-PCR to construct a silkworm gene expression library. Key polyhedrin promoter bait sequences were synthesized to generate a bait yeast strain, which was used to screen the one-hybrid cDNA library.

**Results:**

In total, 12 positive yeast colonies were obtained from the SD/-Leu/AbA plates; sequencing analysis showed that they belong to two different protein cDNA colonies. Positive colonies underwent bioinformatics analysis, which revealed one colony to be ribosomal proteins [*B. mori* ribosomal protein SA (BmRPSA)] and the other to be NPV DNA-binding proteins (DBP). To further verify the regulatory function of these two protein groups, transient expression vectors (pSK-IE-*dbp* and pSK-IE-*BmRPSA*) were constructed. The recombinant plasmids were then transfected into cultured *B. mori* N (BmN) cells, which had been infected with a recombinant bacmid containing the gene encoding luciferase (*luc*). The results showed that overexpression of either *dbp* or *BmRPSA* upregulated the *polh* promoter-driven transcription of *luc* in BmN cells. In addition, *dbp* or *BmRPSA* RNA interference (RNAi) resulted in the downregulation of luciferase reporter expression in BmN cells, demonstrating that DBP and BmRPSA are important for *luc* transcription. EMSA results further confirmed that DBP could directly bind to the conserved single-stranded *polh* promoter region *in intro*. However, EMSA assay also showed that BmRPSA did not bind to this region, precluding a direct DNA association.

**Conclusions:**

Both DBP and BmRPSA are important for *polh* transcription. DBP can regulate *polh* promoter activity by direct binding to the conserved single-stranded *polh* promoter region, BmRPSA may regulate *polh* promoter activity by indirect binding to this region.

## Background

Baculoviruses are large, double-stranded DNA (dsDNA) viruses that replicate only in arthropods, mainly insects [1]. The baculovirus expression vector system (BEVS) is a well-known, feasible, safe and effective technology for the production of recombinant proteins in insect or insect-cultured cells. It has also been recognized as one of the putative four major eukaryotic expression systems [2]. In this system, the insect cells are infected by a virus encoding a desired transgene under the powerful baculovirus polyhedrin promoter, which leads to the production of large amounts of protein. However, the mechanism behind the power of the polyhedrin promoter in gene expression in this system is still unclear [3]. Therefore, it would be informative to elucidate the high-level expression mechanism of the polyhedrin promoter, which not only would provide a new theoretical basis for the transformation of the BEVS, but might also bring economic benefits.

Currently, there are many reports on the factors regulating baculovirus late and very late genes. For example, using transient expression assays, Todd *et al.* identified several late expression factors (LEF) of the *Autographa californica* NPV involved in expression from a late baculovirus promoter [4]. McLachlin *et al.* showed that very late facor-1 (VLF-1) is required for strong expression of the polyhedrin gene [5]. However, there is no clear evidence that this gene is directly involved in *polh* promoter transcriptional regulation. Ghosh *et al.* reported that a host factor, polyhedrin promoter binding protein (PPBP), binds to the transcriptionally important motif AATAAATAAGTATT within the *polh* initiator promoter [6]. When PPBP was mopped out *in vivo* by a plasmid carrying the PPBP cognate sequence present in *transpolh* promoter-driven expression of the luciferase reporter was abolished, demonstrating that binding of PPBP to the *polh* promoter is essential for transcription [7].

The major mechanism of differential gene expression is transcriptional regulation, which is controlled by transcription factors that bind to DNA *cis*-elements located in gene promoters [8]. A simple and highly efficient method of identifying protein–DNA interactions is the yeast one-hybrid system [9-11]. In this system, part of the conservative polyhedrin promoter copies of putative transcription regulatory DNA elements [12,13] is cloned as a tandem repeat upstream of a reporter gene in a reporter vector; the reporter gene is then introduced into the yeast genomic DNA via homologous recombination to generate a bait-specific reporter strain [9]. A one-hybrid silkworm gene expression cDNA library is constructed simultaneously. Potential DNA-binding proteins are expressed as fusion proteins containing the yeast GAL4 transcription activation domain (GAL4 AD). The protein–DNA interactions are screened directly in yeast and interactions between target DNA and the hybrid protein are detected by reporter gene expression [9,11]. In the current study, one host factor and one baculovirus factor were found using this process. The involvement of the obtained polyhedrin promoter binding factors in the transcriptional regulation of the *polh* promoter was identified further by the overexpression or RNA interference (RNAi) of these factors inside BmN cells, which were infected with a recombinant bacmid containing the gene encoding luciferase (*luc*). EMSA results further confirmed that DBP could directly bind to the conserved single-stranded *polh* promoter region *in intro*. These results suggest that a novel transcription factor involved in transcriptional regulation of *polh* promoter.

## Results

### Construction of AD fusion cDNA library for yeast one-hybrid system

The fat body tissue of fifth-instar silkworm larvae that had been infected with BmNPV for 5 days was dissected and used to extract total RNA. Purified and concentrated mRNA was used as the first-strand cDNA template and SMART cDNA was amplified by LD-PCR. CHROMA SPINTM + TE-400 Columns were used to select for DNA molecules ≫400–500 bp.

### Identification of bait yeast strain and testing for AbA^r^ expression

**Figure 1:**
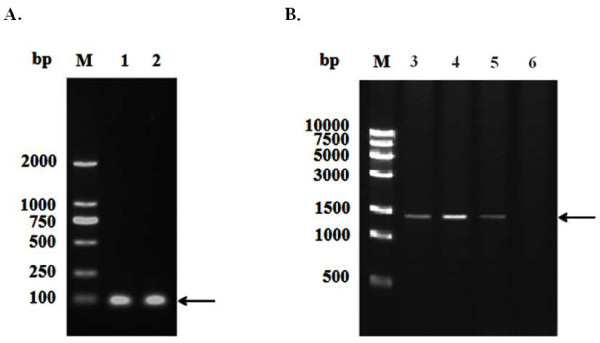
**Identification of the one step PCR products 3rep and 3mut (A) and bait-reporter yeast strain (B).** M, DNA Marker; 1, PCR product of 3rep; 2, PCR product of 3mut; 3, PCR product of Y1HGold (p53-AbAi) reporter yeast strain; 4, PCR product of Y1HGold (p3rep-AbAi) reporter yeast strain; 5, PCR product of Y1HGold (p3mut-AbAi) reporter yeast strain; 6, PCR product of Y1HGold yeast strain. Arrows indicate the target bands.

Based on the conserved sequence of the baculovirus polyhedrin promoter, a three repeated segment (3rep) was designed. In addition, a three repeated mutant segment (3mut) was also designed as a control. The chemical synthesis of a 77 bp-long bait single-stranded (ss)DNA molecule was formed from a dsDNA by one-step PCR (Figure 1A). *Hin*d III and *Xho* I enzymes were then used to release the recombinant 3rep and 3mut to insert into plasmid pAbAi. The recombinant bait plasmid p3rep–AbAi and the mutant plasmid pmut–AbAi were identified by PCR, restriction enzyme digestion and DNA sequencing.

Linearized recombinant plasmid p3rep–AbAi, p3mut–AbAi and p53–AbAi were transformed into *Saccharomyces cerevisiae* Y1HGold. Large healthy colonies were picked and analyzed by PCR. As shown in Figure 1B, the PCR products of yeast bait-reporter Y1HGold (p3rep-AbAi) and Y1HGold (p3mut-AbAi) were 1.425 kb, the PCR products of Y1HGold (p53-AbAi) (positive control) were 1.4 kb. The negative control had no band (Figure 1B).

**Figure 2:**
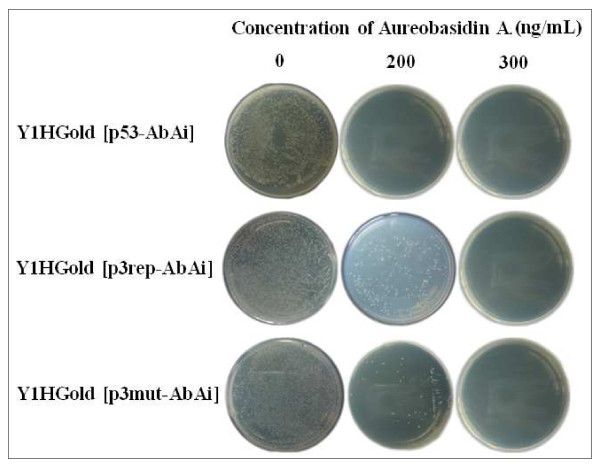
**Testing the bait strain for AbA**^**r**^**expression.** The minimal concentration of Aureobasidin A needed to suppress the basal expression of both Y1HGold (p3rep-AbAi) and Y1HGold (p3mut-AbAi) was 300 ng/mL, whereas for the positive control group [Y1HGold (p53-AbAi)], it was 200 ng/mL.

To omit the influence of the recognition of the target sequence by endogenous yeast transcription factors, the bait stains for AbA^r^ expression were also tested. The testing results of a minimal inhibitory concentration of Aureobasidin A for bait reporter yeast strains are showed in Figure 2. The minimal concentration of Aureobasidin A needed to suppress the basal expression of the Y1HGold (p53-AbAi) yeast strain (the positive control) was 200 ng/mL. The minimal concentration of Aureobasidin A needed to suppress the basal expression of the Y1HGold (p3rep-AbAi) and Y1HGold (p3mut-AbAi]) yeast strains was 300 ng/mL.

### Screening of binding proteins to the polyhedrin core promoter by yeast one-hybrid system

To obtain the proteins interacting with the core elements in the polyhedrin promoter, SMART cDNA and linearized plasmid pGADT7-Rec were transformed into yeast competent cells Y1HGold (p3rep-AbAi) and Y1HGold (p3mut-AbAi), respectively. The cells were then transferred onto agar solidified SD/-Ura and SD/-Ura AbA* media. Competent yeast cells Y1HGold (p53-AbAi) and p53 ragments were used as positive control. In total, approximately 3.0×10^6^ clones were screened to identify 12 positive colonies capable of growing in the present of Aureobasidin A. The plasmids were sequenced in positive clones to reveal that all of them belonged to two types of protein. One encoded DNA-binding proteins (DBP: [GenBank accession number: M63416]) derived from the baculovirus genome, whereas the other encoded a ribosome protein derived from silkworm (BmRPSA: [GenBank accession number: NP_001106143]).

### Identification of the activities of the novel polyhedrin-promoter binding proteins

Analysis of the influence of *dbp* or *BmRPSA* gene transient expression on the activity of the BmNPV polyhedrin gene promoter.

**Figure 3:**
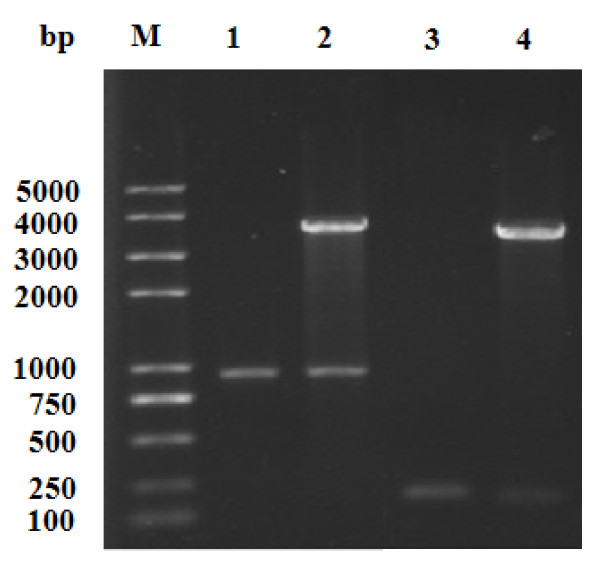
**The identification of recombinant plasmid pSK-IE-*BmRPSA*and pSK-IE-*dbp*.** The *dbp* or *BmRPSA* gene was inserted into the transient expression vector pSK-IE. Recombinant plasmid pSK-IE-*BmRPSA* and pSK-IE-*dbp* were analyzed by PCR and restriction enzyme digestion. M, trans plus II 2000; 1, PCR product of *BmRPSA*; 2, recombinant plasmid digested with *Eco*R I, and *Xho*I; 3, PCR product of *dbp*; 4, recombinant plasmid digested with *Eco*R I, and *Xho* I.

**Figure 4:**
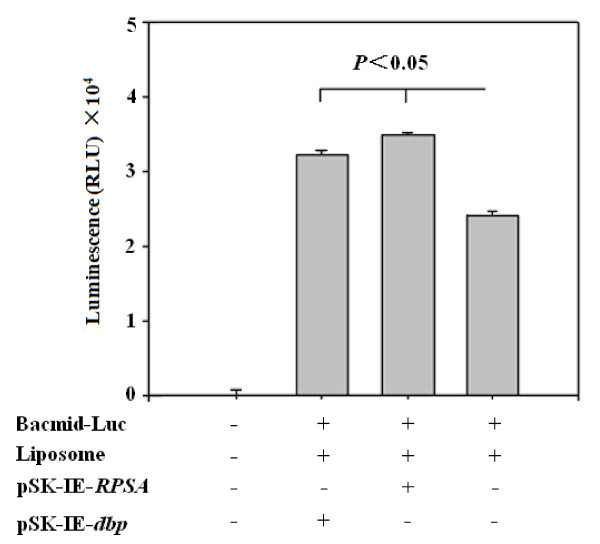
**The influence of the transient expression of*dbp*or*BmRPSA*on the polyhedrin promoter.** Recombinant baculovirus Bacmid-Luc containing *luc* under the control of the polyhedrin gene promoter (as a control) was used to test the effect of *BmRPSA* and *dbp* on *luc* expression. The transient expression vector pSK-IE containing the IE promoter was able to promote the expression of the *BmRPSA* and *dbp* genes. The fluorescence intensity of BmN cells was quantified using a luminometer. Values are expressed as mean TSEM (*n* = 3). Similar results were obtained in three independent experiments.

To determine whether DBP or BmRPSA was required for BmNPV polyhedrin gene promoter activity, two transient expression vectors (pSK-IE-*BmRPSA* and pSK-IE-*dbp*) were constructed (Figure 3) and transformed into BmN cells infected by recombinant virus Bacmid-Luc. Cell lyses were collected 72 h later and chemiluminescence was detected (Figure 4). As shown in Figure 4, compared with the cells infected only by recombinant virus Bacmid-Luc, cell luminescence were significantly enhanced after transfection with the transient expression plasmids pSK-IE-*dbp* or pSK-IE-*BmRPSA,* indicating that overexpression of DBP or BmRPSA could enhance polyhedrin promoter activity.

Analysis of the effects of DBP and BmRPSA on BmNPV polyhedrin gene promoter activity by RNAi assay.

**Figure 5:**
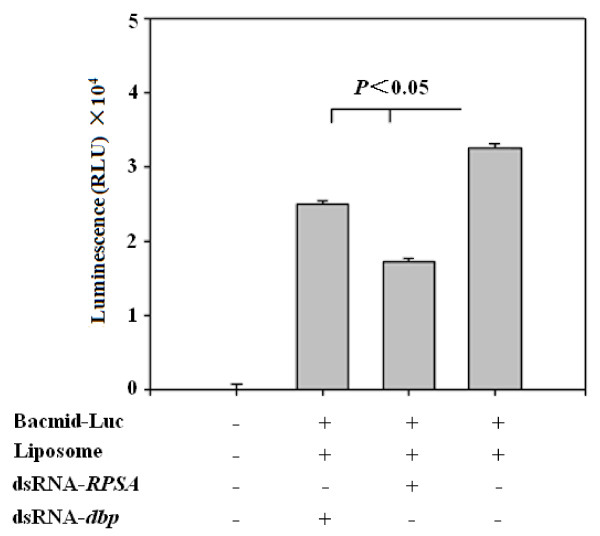
**The RNAi assay of*dbp*and*BmRPSA*.** Recombinant baculovirus Bacmid-Luc containing the luciferase reporter gene under the control of polyhedrin gene promoter (control) was used to test the activities of *BmRPSA* and *dbp* on *luc* expression using an RNAi assay. The fluorescence intensity of BmN cells was quantified using a luminometer. Values are expressed as mean TSEM (*n* = 3). Similar results were obtained in three independent experiments.

To further test the influence of DBP and BmRPSA on BmNPV polyhedrin gene promoter activity, the dsRNA of *dbp* and *BmRPSA* were synthesized and transfected into BmN cells infected by the recombinant virus Bacmid-Luc. After being cultured at 27°C for 2 days, the cells were collected and lysed by lysis buffer. The fluorescence value of the supernatant was determined by the Luciferase Assay. As shown in Figure 5, the chemiluminescence value of the negative control group was very low. The chemiluminescence value was decreased significantly after *dbp* or *BmRPSA* dsRNA transfection compared with cells infected only by recombinant virus Bacmid-Luc (*P* <0.05). These results indicate that downregulation of either *dbp* or *BmRPSA* could decrease polyhedrin promoter activity.

Electrophoretic mobility shift assays (EMSA) analysis of DBP and BmRPSA binding to BmNPV polyhedrin gene promoter *in vitro.*

**Figure 6:**
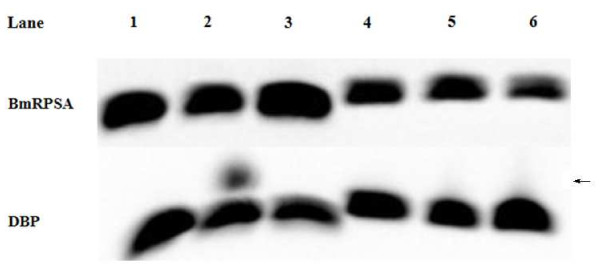
**EMSA analysis of DBP and BmRPSA binding to BmNPV polyhedrin gene promoter*in vitro*.** A biotin-labeled 77 bp-long 3 repeats polyhedrin gene promoter ssDNA and dsDNA fragments were incubated with purified BmRPSA (or DBP) protein, respectively. The mixture was separated by native polyacrylamide gel electrophoresis. 1, biotin-labeled ssDNA; 2, biotin-labeled ssDNA + BmRPSA (or DBP); 3, biotin-labeled ssDNA + BmRPSA (or DBP) + 100 fold molar excess of unlabeled ssDNA; 4, biotin-labeled dsDNA; 5, biotin-labeled dsDNA + BmRPSA (or DBP); 6, biotin-labeled dsDNA + BmRPSA (or DBP) + 100 fold molar excess of unlabeled dsDNA. Arrow indicates the DNA–DBP complex.

To test whether DBP or BmRPSA indeed recognized the motif at the conserved BmNPV polyhedrin gene promoter, in vitro EMSA was performed. A 77 bp-long 3 repeats single-stranded (ss) DNA molecule or double-stranded (ds) DNA from the BmNPV polyhedrin gene promoter (3rep) was biotin-labeled and incubated with purified DBP or BmRPSA proteins. Formation of the DBP–DNA or BmRPSA-DNA complex was detected (Figure 6), the formation of a DBP–DNA complex confirmed that DBP could directly bind to this fragment. However, the binding of BmRPSA to this fragment was not obvious, precluding a direct DNA association.

## Discussion

The BEVS is an efficient tool for the production of recombinant proteins in extremely large quantities. Foreign genes are inserted into the baculovirus genome under the control of the potential polyhedrin promoter to activate the expression of foreign genes; gene expression is regulated by the host or viral transcription factors by binding to the promoter sequence *cis*-elements upstream of polyhedrin gene [14]. Therefore, study of the polyhedrin gene promoter binding protein is essential to clarify the high-level transcription mechanism involved in this regulation.

Yeast one-hybrid screening is widely used for the identification of transcription factors (TFs) that interact with specific DNA sequences [15]. DNA binding sites for TFs and potential binding proteins were identified by the analysis of reporter gene expression in yeast cells. It also the advantage that the encoding nucleotide sequence of DNA-binding proteins is obtained directly from the gene library without the need for complex protein separation or purification. In addition, yeast is a eukaryote and, therefore, the results obtained using the yeast system *in vitro* better reflect the ‘real’ situation within the eukaryotic cell gene [16]. In the yeast one-hybrid system, construction of cDNA AD fusion library for screening is a key step to obtaining *trans*-acting protein factors [17]. Therefore, in the current study, total RNA was extracted from the fat body of infected fifth-instar silkworm larvae 120 h post-infection. Purified mRNA was used to synthesis SMARTer ds cDNA by LD-PCR, which was used to construct a cDNA AD fusion expression library for a one-hybrid yeast system. Using this library to screen BmNPV polyhedrin promoter binding protein will include not only host, but also viral factors.

According to previous reports, the polyhedrin gene promoter sequence is 129 bp long. The major determinant for promoter activity was narrowed to within eight nucleotides, TAAGTATT, at the start point of polyhedrin mRNA transcription. Mutations within TAAGTATT blocked initiation of transcription and resulted in a 2000-times decrease in transcription activity [18]. Mutation immediately upstream (nt −52 to −60) from the transcription start point reduced expression four-times [19]. Therefore, we designed a 77-bp tandem repeated segment of the conserved polyhedrin gene promoter region (nt −44 to −60) synthesized by chemical synthesis. The tandem repeated segment was integrated into the Y1HGold genome to create a bait strain, which was used to screen a cDNA AD fusion expression library. Through repeated screening, two positive factor binding proteins (BmRPSA and DBP) were found. The RPSA, previously named 37-kDa laminin receptor precursor/67-kDa laminin receptor (LRP/LR) [20,21], is a multifunctional protein that has a role in several pathological processes. It is required for the assembly and stability of the 40 S ribosomal subunit and the processing of the 20 S rRNA-precursor to mature 18 S rRNA in a late step of the maturation of 40 S ribosomal subunits [22]. Terranova *et al.* found that it functions as a cell surface receptor for laminin and also has a role in cell adhesion to the basement membrane and in the consequent activation of signaling transduction pathways. It also acts as a receptor for several other ligands, including the pathogenic prion protein, viruses and bacteria [23,24]. Baculovirus DBP was first described after being purified from BmNPV-infected cells and subsequently shown to have properties of a bone fide ssDNA-binding protein, although its role in virus replication is unclear [25].

However, few reports have revealed whether BmRPSA and DBP are involved in baculovirus late gene expression regulation. To determine the possible functions of BmRPSA and DBP in BmNPV polyhedrin gene promoter activity, we constructed a recombinant BmNPV bacmid-Luc, in which the luciferase reporter gene was under the control of polyhedrin promoter; transient expression and RNAi assays were then performed to characterize the regulation abilities of BmRPSA and DBP to the expression level of the luciferase reporter gene in BmN cells. A transient expression vector pSK-IE containing the IE promoter was used to construct the transient expression vectors pSK-IE-*BmRPSA* and pSK-IE-*dbp*. This vector can be used for both the transient and stable expression of genes [26]. In addition, dsRNA of *BmRPSA* and *dbp* was synthesized and transfected into BmN cells, which were then infected with the recombinant virus Bacmid-Luc. Chemiluminescence detection results showed that the fluorescence intensity of BmN cell lysis was affected by the up- or downregulation of *BmRPSA* and *dbp* gene expression. EMSA results showed that only DBP could directly bind to conserved single-stranded *polh* promoter *in intro*, but the binding of BmRPSA to the *polh* promoter was not obvious. These results suggest that baculovirus DBP is involved in regulating *polh* promoter activity by direct binding to the conserved *polh* promoter region, which is consistent with previous study [25], and the host-derived BmRPSA may regulate *polh* promoter activity by indirect binding to this region. Moreover, up- or downregulation of gene expression is controlled by gene transcription complexes and involves BmRPSA and DBP; it might also involve several additional factors, some of which might interact directly with BmRPSA or DBP. To this end, using a proteomics approach to identify candidates that interact with BmRPSA and DBP could provide significant insight into the roles of BmRPSA and DBP during baculovirus late gene expression. The BEVS is now widely used for recombinant protein production in insect or insect-derived cells, BmRPSA and DBP proteins could be effectively used to promote the recombinant protein expression level by binding with the polyhedrin promoter and it might bring economic benefits.

## Conclusions

In this study, two novel transcriptional regulators, DBP and BmRPSA, were successfully screened from the silkworm gene expression cDNA library. Overexpression and RNAi assay results showed that both DBP and BmRPSA were important for *polh* transcription. EMSA results further confirmed that only DBP could directly bind to the conserved single-stranded *polh* promoter region *in intro*. These results showed that DBP could regulate *polh* promoter activity by direct binding to the conserved single-stranded *polh* promoter region, BmRPSA might regulate *polh* promoter activity by indirect binding to this region

## Materials and methods

### Materials

*Escherichia coli* strains TG1 and DH5α and BmN cells derived from *Bombyx mori* were maintained in our laboratory; pBluescript SK plus plasmid was stored in our laboratory. Recombinant virus Bacmid-Luc containing the luciferase reporter and the transient expression vector pSK-IE were also constructed and stored in our laboratory. The following kits and reagents were used: Matchmaker™ Gold Yeast One-Hybrid Library Screening System Kit, Matchmaker Insert Check PCR Mix 1, LB medium, YPDA medium and SD medium (Clontech, Mountain View, CA, USA); RNAlater™ (RNA stabilization solution) (Qiagen, Germantown, MD, USA); TRIzol kit® and M-MLV reverse transcriptase (Invitrogen, Carlsbad, CA, USA); PolyATtract® mRNA Isolation System III, T7 RiboMAX™ Express RNAi System and Luciferase Assay System (Promega, Madison, WI, USA); LightShift® Chemiluminescent EMSA Kit (Pierce, Rockford, IL, USA); plasmid vector pMD-18 T, T4 DNA ligase, restriction endonuclease *Xho* I, *Eco*R I and *Hin*d III (Takara, Osaka, Japan ); restriction endonuclease *Bbs*I, Taq and related reagents (Fermentas, Burlington, USA);primers, BmNPV promoter core conservative three repeated segment (3rep) and its mutant segment (3mut) were synthesized by Sangon Biotech (Shanghai, China) Co., Ltd. DNA sequencing was completed by Sunny Biotech (Shanghai, China) Co., Ltd.

### Construction of complementary DNA (cDNA) AD fusion library for the yeast one-hybrid system

#### Extraction of total RNA from fat body of silkworm larvae

After sterilization of the skin using 75 % ethanol, the fifth-instar silkworm larvae that had been infected with *B. mori* nuclear polyhedrosis virus BmNPV for 5 days, were dissected in RNA Stabilization Solution to separate the fat body from the rest of the larva. Tissue (100 mg) was pulverized in liquid nitrogen using a mortar and pestle. The powered tissue was then transferred to a centrifuge tube containing 1 ml TRIzol. The total RNA was then extracted from the larval tissue. The TRIzol kit was used according to the manufacturer’s instructions.

### Purification of mRNA from silkworm fat body total RNA

The PolyATtract® mRNA Isolation System III was used to purify mRNA from total RNA, according to the manufacturer’s instructions. The purified mRNA was condensed by adding 1/10 volume of 3 M sodium acetate, pH 5.2, and an equal volume of isopropanol at −20°C for 1 h; the solution was then centrifuged at 16 000×g for 30 min. After removal of the supernatant, 1 ml 75 % ethanol was added to resuspend the pellet. The solution was then centrifuged at 16 000×g for 15 min. The mRNA was resuspended in 100 μL of DEPC treated-water.

### Synthesis of first-strand SMART cDNA

CDS (oligo-dT) primer (1 μL) and 2 μL deionized H_2_O were added to 1 μL of purified and quantitative mRNA (0.025–1.0 μg poly A+). After incubation for 2 min at 72°C, the mixture was transferred on ice and the following reagents were added: 5×First-Strand Buffer 2 μL, DTT (100 mM) 1 μL, dNTP mix (10 mM) 1 μL and SMART MMLV RT 1 μL. The sample was incubated at 42°C for 10 min, and then at 42°C for another hour after the addition of 1 μL BD SMART III Oligo. After the sample was placed at 75°C for 1 min to terminate the synthesis, 1 μL RNase H (2 units) was added and the sample was then incubated at 37°C for 20 min. The final product was stored at −20°C.

### Amplification of SMART cDNA by long-distance PCR (LD-PCR)

The following reagents were combined and mixed for PCR amplification: 2 μL first-strand SMART cDNA synthesized as above, 70 μL deionized water, 10 μL 10×Advantage 2 PCR Buffer, 2 μL 50 × dNTP Mix, 2 μL 5′ PCR Primer, 2 μL 3′ PCR Primer,10 μL Melting Solution and 2 μL 50×Advantage 2 Polymerase Mix. The cycling parameters were as follows: 95°C for 30 s; 26 cycles of 95°C for 10 s and 68°C for 6 min (the cycler was programmed to increase the extension time by 5 s with each successive cycle); followed by 68°C for 5 min. PCR products were subsequently detected by agarose gel electrophoresis and stored at −20°C.

### Purification of ds cDNA

A BD CHROMA SPIN ^TM^-400 Column was used to purify ds cDNA to remove any small fragments. 1/10 volume of 3 M NaAc and 2.5 volume of 95 % ethanol were added to precipitate the sample at −20°C for 1 h. After centrifugation and removal of the supernatant, the pellet was then washed with 75 % ethanol twice, dried and dissolved in sterile water (100–150 ng/μL). The final product was stored at −20°C.

### Generation of yeast reporter strains for one-hybrid screening

#### Design of target sequence trimer bait

According to pervious references [18,19], the *B. mori* baculovirus polyhedrin promoter contains a highly conserved domain (rep: GCAAATAAATAAGTATT). We therefore prepared a DNA fragment ‘3rep’ comprising three tandemly iterated copies of the highly conserved element. In addition, three tandemly iterated copies of a mutant element were also designed as a control. A *Hin*d III site and a *Xho* I site were introduced before the start codon and after the stop codon, respectively. Table 1 lists the *cis*-elements. One-step PCR was used to synthesize the dsDNA and the reactions were performed at 95°C for 5 min, followed by 68°C for 30 min. The synthesized segments were recycled by PCR kit. Detailed methodology is given in the Promega WizardR SV Gel and PCR Clean-Up System manual.

**Table 1:** **Oligonucleotides used for the generation of bait sequences**^**a**^

***Cis*-element repeat**	**Primer sequence**
Tandem repeat segments (3rep)	5′-CCGAAGCTTGCAAATAAATAAGTATTGCAAATAAATAAGTATT GCAAATAAATAAGTATTCTCGAGGCCATGCCAT-3′
3rep antisense	5′ -ATGGCATGGCCTCGAGAATAC-3′
Tandem repeat mutant segments (3mut)	5′-CCGAAGCTTATGGGTGGGTGGATGTTTACTTTCAGACTTGAGTT ACTTTCAGACTTGAGTTCTCGAGGCCATGCCAT-3′
3mut antisense	5′ -ATGGCATGGCCTCGAGC-3′

^a^Underlined sequences represent several repeats of the polyhedrin promoter conserved sequence ‘rep’ *cis*-elements and the mutant ‘3mut’. Letters in boxes represent the restriction enzymes *Hin*d III and *Xho* I.

#### *Construction of recombinant bait plasmid*

The purified PCR products 3rep and 3mut were inserted into the T vector plasmid pMD 18 T. The recombinant plasmids were digested with *Hin*d III and *Xho* I and then inserted into plasmid pAbAi. The DNA sequences of recombinant bait plasmid p3rep–AbAi and the mutant plasmid p3mut–AbAi were identified by DNA sequencing. After digestion by *Bbs* I, the recombinant plasmids were then purified using the Promega PCR kit and stored at −20°C.

### Transformation of linearized bait plasmids into yeast cells

*Bbs* I linearized recombinant bait plasmid (2 μg) of and 5 μL Yeast Carrier DNA were added to 50 μL yeast competent cells treated with LiAc. After incubation at 30°C for 30 min, the competent cells were mixed with 20 μL DMSO. The cells were then heat shocked for 15 min at 42°C and then further centrifuged to discard the supernatant; the pellet was resuspended with 1 ml 0.9 % NaCl. 100 μL transformed competent cells were then transferred onto agar solidified SD/-Ura media. The media was incubated at 30°C for 3–5 days. Linearized mutant plasmid p3mut–AbAi was used as negative control, whereas linearized plasmid p53–AbAi was used as positive control (provided by Matchmaker^TM^ Gold Yeast One-Hybrid Library Screening System Kit).

### *PCR identification of yeast bait-reporter*

Single clones of Y1HGold (p3rep–AbAi) yeast strain, Y1HGold (p53–AbAi) yeast stains and Y1HGold (p3mut–AbAi) yeast stains were picked and cultured overnight at 30°C for PCR identification. The components of the reaction mixture were as follows: 25 μL recombinant yeast stains and 25 μL Matchmaker Insert Check PCR Mix. The PCR reaction was performed using 30 cycles of denaturation at 98°C for 10 s, primer annealing at 55°C for 30 s and extension at 68°C for 2 min. The expected size of PCR products were as follows: positive control, 1.4 kb; negative control, no band; bait strain, 1.35 kb plus the inserted fragment.

### Evaluation of yeast bait-reporter for AbA^r^ expression

To test the antibiotic system, healthy yeast colonies of each group were picked and resuspended with 0.9 % NaCl (for approximately 2000 cells per 100 μL); 100 μL yeast strain was transferred onto each of the following agar solidified medias: SD/-Ura, SD/-Ura with AbA(100 ng/mL), SD/-Ura with AbA(200 ng/mL) and SD/-Ura with AbA(300 ng/mL). The colonies were allowed to grow for 2–3 days at 30°C.

### Screening of binding proteins using the yeast one-hybrid system

Yeast competent cells Y1HGold (p53-AbAi), Y1HGold (p3rep-AbAi) and Y1HGold (p3mut-AbAi) were prepared as described previously. 600 μL competent cells Y1HGold (p3rep-AbAi), 2.5 mL PGE/LiAc and 3 μg of pGADT7-Rec (*Sma* I,-linearized) were added to 2–5 μg ds cDNA synthesized by SMART PCR in section 1.2.1. Meanwhile, 50 μL competent cells Y1HGold (p53-AbAi), 100 ng p53 fragment and 1 μg pGADT7-Rec (*Sma* I,-linearized) were added to the positive control group. 600 μL competent cells Y1HGold (p3mut-AbAi), 2.5 mL PGE/LiAc and 3 μg pGADT7-Rec (*Sma* I,-linearized) were added to the negative control group. The competent cells were then incubated at 30°C for 45 min, followed by heat shock at 40°C for 30 min after the addition of DMSO. After centrifuged at 700 g for 5 min to discard the supernatant, the cells were resuspended with 3 mL YPDA medium. The cells continued to be shaken at 250 rpm for 90 min at 30°C. After centrifuged at 700 g for 5 min, the cells were resuspended in 3 mL 0.9 % NaCl. 100 μL yeast culture was transferred onto agar solidified SD/-Ura media and SD/-Ura AbA* media (* refers to the concentration of AbA used for selection). The plates were incubated at 30°C for 3–5 days. SD/-Ura AbA^200^ was used for the p53 control reaction. The screened yeast colony monoclone was then transferred onto SD/-Leu/AbA* for repeated screening. After being incubated for 2–4 days, the positive clone was the one that was single and healthy. The single positive clone was isolated, and the nucleotide sequence of the isolated cDNA determined by DNA sequence.

### Function analysis of binding protein

#### Bioinformatics analysis of BmRPSA and DBP

Analysis of the nucleotide sequence and protein homology of BmRPSA and DBP were performed by related programs or software on NCBI (http://www.ncbi.nlm.nih.gov).

### Transient expression assay of *BmRPSA* and *dbp*

For the transient expression assay, the full-length coding regions of the *BmRPSA* and *dbp* genes were amplified by RT-PCR. The PCR products were then cloned into the *Eco*R I, and *Xho* I, digested pSK-IE vector containing the IE promoter to generate the BmN transient expression vectors pSK-IE-*BmRPSA* and pSK-IE-*dbp*. A liposome-encapsulated transient expression vector was transformed into BmN cells seeded in 12 welled plates (1×10^5^ cells/well) and cultured at 27°C for 48 h; the cells were then infected with the recombinant virus Bacmid-Luc. The infected cells continued to be cultured at 27°C for 48 h and were then collected and the fluorescence intensity measured immediately using a luminometer (Tecan, Switzerland). The excitation wavelength was 488 nm and the emission wavelength was 530 nm. Normal cells were used as a blank control, whereas the cells transfected by the equivalent liposome and Bacmid-Luc were negative controls.

### RNA interference assays of *BmRPSA* and *dbp*

#### Synthesis of *BmRPSA* and *dbp* dsRNA

To obtain the synthesized dsRNA template *in vitro*, the target gene cDNA (*BmRPSA* or *dbp*) was inserted downstream of the T7 promoter from a forward or reverse direction, respectively. Thus, two forward primers (Pa and Pb) containing the T7 promoter and two reverse primers (P1 and P2) containing *BmRPSA* were synthesized (Table 2). The first primer pair (Pa and P2) and the second primer pair (Pb and P1) were used to generate forward and reverse templates for *BmRPSA* dsRNA synthesis, respectively, whereas the recombinant plasmid pGADT7-Rec-*BmRPSA* was used as a template. Meanwhile, two forward primers (Pc and Pd) containing the T7 promoter and two reverse primers (P3 and P4) of *dbp* were also synthesized (Table 2). The first primer pair (Pc and P3) and the second primer pair (Pd and P4) were used to generate forward and reverse templates for *dbp* dsRNA synthesis, respectively, whereas the recombinant plasmid pGADT7-Rec-*BmRPSA* was used as a template. Following the Promega T7 RiboMAX™ Express RNAi System manual, dsRNA was synthesized and its concentration determined (OD_260_×40×dilution ratio μg/mL).

**Table 2:** **Underlined sequences represent T7 promoter and letters in boxes represent restriction enzyme*Eco*RI and*Xho*I**

**Primer**	**Primer sequence**
Pa	5′-GGATCCTAATACGACTCACTATAGGATGTCGGGAGGATTAGACGT-3′
Pb	5′ GGATCCTAATACGACTCACTATAGGTTATGCAGCACTCCAGTCTT-3^’^
P1	5′-CCGGAATTCATGTCGGGAGGATTAG-3'
P2	5'-CCGCTCGAGTTATGCAGCACTCCAG-3'
Pc	5'-GGATCCTAATACGACTCACTATAGGATGGTTTATCGTCGCCGTCG-3'
Pd	5'-GGATCCTAATACGACTCACTATAGGTTAATAGTAGCGTGTTCTGT-3'
P3	5'-CCGGAATTCATGGTTTATCGTCGCC-3'
P4	5'-CCGCTCGAGTTAATAGTAGCGTGTTCT-3'

### Transfection of dsRNA

Successful transfection of long dsRNA into mammalian oocytes has been described elsewhere [27]. This experiment used BmN cells seeded in 12 welled-plates (1×10^5^ cells/well), which were transfected with purified dsRNA (30 ng/μL), using liposomes (35 μg/mL) in sf-900 media. After being cultured at 27°C for 1.5 days, the cells were infected by recombinant virus Bacmid-Luc at MOI = 10. The infected cells continued to be cultured at 27°C for 48 h. After pathogenesis of the virus, the cells were collected, washed with PBS and resuspended in lysis buffer. The pyrolysis products were further centrifuged at 12 000 g for 15 s and the supernatant was added to a 96 welled plate mixed with 100 μL Luciferase Assay Reagent. The fluorescence value was determined immediately (method is as referred to above). Normal cells were used as a blank control, whereas cells transfected by the equivalent liposome and Bacmid-Luc were used as a negative control.

### Preparation of recombinant DBP, BmRPSA and EMSA analysis

BmN cells were grown as described above. *BmRPSA* or *dbp* fused with 6 × His was inserted into transient expression vector pSK-IE, respectively. The liposome-encapsulated transient expression vector was transformed into BmN cells to express the target proteins. The purification of His-DBP protein and His-BmRPSA were performed following standard molecular biology protocols. EMSAs were performed with either 100 ng of recombinant proteins and Biotin-labeled ssDNA (or dsDNA). Biotin-labeled ssDNA (or dsDNA) was used as a control. Protein-DNA complexes were resolved by gel electrophoresis on 6 % non-denaturing polyacrylamide gels in 0.5 × TBE buffer at room temperature. The Biotin-labeled DNA was detected by Chemiluminescence after electrophoresis.

## Competing interests

All authors declare they have no actual or potential competing financial interest.

## Authors’ contributions

WY participated in the design of the study and carried out the RNAi assay, transient expression assay and drafted the manuscript. JL carried out the RNAi assay, transient expression assay, the construction of the cDNA library and participated in the screening of binding proteins. MW carried out the EMSA analysis. YQ and ZN participated in the sequence alignment and performed the statistical analysis. JC participated in RNAi assay. ZL and YZ helped to draft the manuscript. All authors read and approved the final manuscript.
